# Tumor Staging of Endocervical Adenocarcinoma: Recommendations From the International Society of Gynecological Pathologists

**DOI:** 10.1097/PGP.0000000000000758

**Published:** 2021-02-09

**Authors:** Kay J. Park, Andres Roma, Naveena Singh, C. Blake Gilks, Esther Oliva, Nadeem Abu-Rustum, Pedro T. Ramirez, W. Glenn McCluggage

**Affiliations:** Department of Pathology (K.J.P.); Department of Surgery, Gynecologic Oncology Division (N.A.-R.), Memorial Sloan Kettering Cancer Center, New York, New York; Department of Pathology, University of California San Diego, San Diego, California (A.R.); Department of Cellular Pathology, Barts Health NHS Trust, London (N.S.); Department of Pathology, Belfast Health and Social Care Trust, Belfast (W.G.M.C.), UK; Department of Pathology and Laboratory Medicine, University of British Columbia, Vancouver, British Columbia, Canada (C.B.G.); Department of Pathology, Massachusetts General Hospital, Boston, Massachusetts (E.O.); Department of Gynecologic Oncology and Reproductive Medicine, Division of Surgery, MD Anderson Cancer Center, Houston, Texas (P.T.R.)

**Keywords:** Endocervical adenocarcinoma, FIGO, TNM, Staging

## Abstract

The International Federation of Gynecology and Obstetrics (FIGO) updated its staging system for cervical cancer in 2018 with changes that affect size criteria for early stage disease, as well as including pathology and radiology in addition to clinical assessment to be used in staging. Lymph node involvement was also included in the staging system. In early stage disease, pathologic findings are crucial in determining stage, which in turn determine treatment and prognosis for the patient. Therefore, it is imperative that there are unified and consistent methods and recommendations for assessing and reporting pathologic parameters for accurate staging. We describe the changes in the revised FIGO staging scheme and discuss controversial issues in cervical cancer staging from a pathologic perspective. We also provide practical recommendations regarding these parameters based on literature review and/or expert opinion/consensus.

Accurate tumor staging is important for patient prognostication and optimal management, and also has wider implications for international benchmarking, cancer registration and research, and provides an invaluable epidemiological resource. There are several controversial issues in pathologic staging of cervical carcinomas in general and some specific to adenocarcinomas, as borne out by the International Society of Gynecological Pathologists (ISGyP) survey results published elsewhere in this issue (McCluggage). These include multiple issues with regard to measuring tumor size, particularly in patients with multifocal tumors, differences in gross and microscopic tumor size, purely exophytic tumors, clinically visible but small tumors and shallow but wide tumors. Other controversial topics include assessment of parametrial/paracervical involvement, adnexal involvement, and reporting of lymph node involvement; there are also related elements such as quantifying lymphovascular invasion which are not part of the current staging system. These all obviously have important implications for accurately staging cervical cancers. We hereby aim to provide recommendations based on published literature and sometimes expert opinion and consensus. Many of these are also detailed in the International Collaboration on Cancer Reporting (ICCR) data set for reporting of carcinomas of the cervix 1.

## INTERNATIONAL FEDERATION OF GYNECOLOGY AND OBSTETRICS (FIGO) 2018 STAGING SYSTEM FOR CERVICAL CANCERS

In 2018, the FIGO revised the staging system for carcinoma of the uterine cervix, for the first time allowing incorporation of imaging and/or pathologic findings in the assessment of tumor size and disease extent 2. There were a number of reasons for this according to FIGO, including the technological advances in imaging modalities, and the fact that the pathological findings were sometimes not concordant with clinical staging, leading to an increase in para-aortic lymph node sampling to determine the need for extended field radiation.

The major changes to the revised FIGO 2018 staging include (1) the removal of the 7 mm horizontal extent as a parameter for stage IA carcinoma; (2) dividing stage IB into 3 subgroups (IB1, IB2, IB3); (3) including lymph node involvement in stage assignment (stage IIIC1 with only pelvic lymph node involvement, stage IIIC2 with para-aortic nodal involvement); (4) allowing the use of imaging to assess lymph nodes and (5) denoting “p” for pathologic or “r” for radiologic to indicate the method used to derive the stage. The intent was to allow the staging system to be applicable to all resource levels, including low- and middle-income countries, acknowledging the concern that access to imaging modalities or surgico-pathologic documentation of disease extent may not always be feasible.

Unfortunately, there were errors in the original publication of the new FIGO staging in the *International Journal of Gynecology and Obstetrics*. The “=” in measurement cut-offs was erroneously placed in 12 instances in stages I and II (Table 1), and there were also several statements that were confusing or vague. These were pointed out by users and in particular by members of the ICCR data set authoring committee on cervical cancer reporting. As a result, a corrigendum was subsequently published with corrections 2,3. The key amendments to the staging are delineated in Table 2, and the FIGO response to most of the queries raised by ICCR are listed in Table 3. A recent manuscript by Salvo et al. 4 highlights various flaws in the revised FIGO 2018 staging and particularly points to the gaps in pathologic evaluation of cervical cancers.

**TABLE 1:** Cervical cancer staging FIGO 2009 compared with FIGO 2018

FIGO 2009	FIGO 2018
Stage I: The carcinoma is strictly confined to the cervix uteri (extension to the corpus is disregarded) IA Invasive carcinoma that can be diagnosed only by microscopy with deepest invasion ≤5 mm and largest extension ≤7 mm IA1 Measured stromal invasion ≤3 mm in depth and extension ≤7 mm IA2 Measured stromal invasion >3 mm and not >5 mm with extension not >7 mm IB Clinically visible lesions limited to cervix or preclinical cancers greater than stage IA* IB1 Clinically visible lesion ≤4 cm in greatest dimension IB2 Clinically visible lesion >4 cm in greatest dimension	Stage I: The carcinoma is strictly confined to the cervix uteri (extension to the corpus is disregarded) IA Invasive carcinoma that can be diagnosed only by microscopy, with maximum depth of invasion ≤5 mm† IA1 Measured stromal invasion ≤3 mm in depth IA2 Measured stromal invasion >3 mm and ≤5 mm in depth IB Invasive carcinoma with measured deepest invasion >5 mm (greater than stage IA); lesion limited to the cervix uteri with size measured by maximum tumor diameter‡ IB1 Invasive carcinoma >5 mm depth of stromal invasion and ≤2 cm in greatest dimension IB2 Invasive carcinoma >2 cm and ≤4 cm in greatest dimension IB3 Invasive carcinoma >4 cm in greatest dimension
Stage II: Cervical carcinoma invades beyond uterus, but not to pelvic wall or lower third vagina IIA Without parametrial invasion IIA1 Clinically visible lesion ≤4 cm in greatest dimension IIA2 Clinically visible lesion >4 cm in greatest dimension IIB With obvious parametrial invasion	Stage II: The cervical carcinoma invades beyond the uterus, but has not extended onto the lower third of the vagina or to the pelvic wall IIA Involvement limited to the upper two-third of the vagina without parametrial invasion IIA1 Invasive carcinoma ≤4 cm in greatest dimension IIA2 Invasive carcinoma >4 cm in greatest dimension IIB With parametrial invasion but not up to pelvic wall
Stage III: The tumor extends to the pelvic wall and/or involves lower third of vagina and/or caused hydronephrosis or nonfunctioning kidney IIIA Tumor involves lower third of vagina with no extension to pelvic wall IIIB Extension to pelvic wall and/or hydronephrosis or nonfunctioning kidney	Stage III: The carcinoma involves the lower third of the vagina and/or extends to the pelvic wall and/or causes hydronephrosis or nonfunctioning kidney and/or involves pelvic and/or para-aortic lymph nodes IIIA Carcinoma involves lower third of the vagina, with no extension to the pelvic wall IIIB Extension to the pelvic wall and/or hydronephrosis or nonfunctioning kidney (unless known to be due to another cause) IIIC Involvement of pelvic and/or para-aortic lymph nodes (including micrometastases)§, irrespective of tumor size and extent (with r and p notations)∥ IIIC1 Pelvic lymph node metastasis only IIIC2 Para-aortic lymph node metastasis
Stage IV: The carcinoma has extended beyond the true pelvis or has involved (biopsy proven) the mucosa of the bladder or rectum. A bullous edema, as such, does not permit a case to be allotted to Stage IV IVA Spread of the growth to adjacent organs IVB Spread to distant organs	Stage IV: The carcinoma has extended beyond the true pelvis or has involved (biopsy proven) the mucosa of the bladder or rectum. A bullous edema, as such, does not permit a case to be allotted to stage IV IVA Spread of the growth to adjacent organs IVB spread to distant organs

* All macroscopically visible lesions—even with superficial invasion—are allotted to stage IB carcinomas.

† Imaging and pathology can be used, when available, to supplement clinical findings with respect to tumor size and extent, in all stages. Pathological findings supersede imaging and clinical findings.

‡ The involvement of vascular/lymphatic spaces should not change the staging. The lateral extent of the lesion is no longer considered.

§ Isolated tumor cells do not change the stage but their presence should be recorded.

∥ Adding notation of r (imaging) and p (pathology) to indicate the findings that are used to allocate the case to stage IIIC. For example, if imaging indicates pelvic lymph node metastasis, the stage allocation would be Stage IIIC1r; if confirmed by pathological findings, it would be stage IIIC1p. The type of imaging modality or pathology technique used should always be documented. When in doubt, the lower staging should be assigned.

**TABLE 2:** Key amendments to FIGO 2018 staging

Allowing use of any imaging and/or pathologic findings for allocating stage (previously only clinical exam)
Stage I Amendments to microscopic pathologic findings and size designation Allowing use of imaging and/or pathologic assessment of tumor size
Stage II Allowing use of imaging and/or pathologic assessment of size and extent
Stage III Including nodal involvement as part of staging Allowing assessment of retroperitoneal lymph nodes by imaging and/or pathologic findings and if deemed metastatic the case is designated as IIIC1 (pelvic/parametrial LN+) or IIIC2 (para-aortic LN+) with notation of method used for stage allocation
No recommendations for routine investigations, which are to be decided on the basis of clinical findings and standard of care. The revised staging system does not mandate the use of a specific imaging technique, lymph node biopsy or surgical assessment of tumor. In low-resourced conditions, clinicians can continue to assess clinically as before.
The method by which tumor is measured should be recorded (r/radiology, p/pathology)

**TABLE 3:** Queries by ICCR to FIGO with response

FIGO 2018 queries by ICCR	FIGO response
“Clinically visible lesions, and those with larger dimensions, are allocated to stage IB”	There is flexibility in how tumors are staged (clinically, radiographically, pathologically); if available, pathologic stage is used; if radiology and pathology are not available, clinical visibility is used to assign stage IB
“The margins should be reported to be negative for disease. If the margins of the cone biopsy are positive for invasive cancer, the patient is allocated to stage IB1”	In the event that the margins of a cone/loop biopsy are positive for the disease, a repeat cone/loop biopsy is required to stage the patient
“The presence of micrometastasis (MIC) or isolated tumor cells (ITCs) may be recorded but their presence does not change the stage”	This was an error in the original publication and the correction has now been made that micrometastases (but not ITCs) constitute stage IIIC
“Presently ovarian involvement does not change the stage.”—Why does this form of extrauterine involvement not change the stage?	Ovarian involvement “does not change stage because of low incidence in early stage disease (<1% in SCC, <5% in other types), often associated with other high risk features, and limited data on impact on survival as an independent risk factor (commentary under stage II section)
Shallow but wide (>7 mm) tumors—what are the data for designating stage by depth of invasion only?	There is not adequate information on prognostic implications of a wide shallow lesion. In cases with multifocal lesions also, there is limited information regarding the impact. Depth correlates best with lymphovascular space invasion and blood vessel invasion because of tumor dislodgement and proximity to blood vessels. Hence depth of invasion is the most important to be noted

FIGO indicates International Federation of Gynecology and Obstetrics; ICCR, International Collaboration on Cancer Reporting.

## WHICH TUMOR STAGING SYSTEM TO USE?

In the cervix, as in other gynecologic organs, both FIGO and TNM [Union for International Cancer Control (UICC) or American Joint Committee on Cancer (AJCC)] staging systems exist. With regard to updating of staging systems, there is collaboration between FIGO and those agencies responsible for TNM with an agreement to adopt FIGO staging but there is no coordination of timing of revisions and generally following the introduction of a new FIGO staging system, this is incorporated into TNM (both UICC and AJCC versions) at a later date. Apart from minor discrepancies in terminology, the UICC and AJCC systems are broadly concurrent.

**Table:** 

Recommendations for staging
Stage should be included in pathology reports as pathologic stage only based on all available pathologic material The staging system used (FIGO 2018, TNM/UICC or both) depends on local practice as some institutions require College of American Pathologists (CAP) AJCC reporting (pT, pN, pM). The staging system used should be indicated alongside the assigned stage

## MEASURING TUMOR SIZE

Tumor size is an important parameter in tumor staging which dictates treatment and patient prognosis. There are several difficult issues regarding tumor size measurements that pathologists often face.

- With a grossly visible tumor, which measurements should be reported and used for staging purposes? Macroscopic, microscopic or a combination?
- Opening a surgical specimen of the cervix longitudinally may demonstrate a maximum dimension that is not evident clinically or radiologically, especially in tumors which circumferentially involve the cervix. Should this measurement supersede the maximum tumor diameter if this is the greater of the 2 measurements?
- In cases with multiple specimens containing tumor (loop(s), cone(s), trachelectomy, hysterectomy), how does one incorporate the measurements from the various specimens to give the most accurate size?
- How does one measure “depth of invasion” in purely or mostly exophytic tumors with no or limited stromal infiltration?
- How should multifocal carcinomas be measured and reported?
- How many dimensions of the tumor should be reported?
- What is meant by the term microinvasive carcinoma?
- In lesions composed of an admixture of adenocarcinoma *in situ* (AIS)/cervical glandular intraepithelial neoplasia (CGIN, terminology used in United Kingdom and some other jurisdictions) and adenocarcinoma, it can be difficult to delineate invasive from noninvasive tumor. In these cases, should the whole lesion be measured, or an attempt made to separate only the invasive component?
- When tumor is present at the margin of a loop or cone excision, how should the tumor be staged?

### Measuring Grossly Visible Tumors

The purpose of providing tumor measurements is ultimately to accurately stage for treatment planning and prognostication. Therefore, whatever measurements are provided should give the treating physician enough information without adding extraneous information. In the 2009 FIGO staging, grossly visible tumors were automatically allotted to stage IB, regardless of tumor size or depth of invasion. This led to neoplasms being upstaged inappropriately in some small tumors and those with only superficial invasion. The revised 2018 FIGO system now states that although a tumor is clinically visible, final stage should be based on all the information available at the time, which includes pathology and radiology, with pathologic findings being the ultimate arbiter of stage. In those situations where pathology and radiology are not available clinical examination should be used to stage tumors. Therefore, we recommend using all available information to best determine true tumor size, which pathologically may require a combination of gross and microscopic measurements. Separate pathologic gross and microscopic measurements *should not be provided* but a single set of measurements based on a combination of gross and microscopic examination; in some cases, gross examination may be more important (for example in larger neoplasms), while in others microscopic examination is more important and many smaller tumors can only be measured microscopically since they are not grossly visible.

**Table:** 

Recommendation for tumor measurement: grossly visible tumors
Use all available material to best assess tumor size, which may require combined gross and microscopic measurements—do not provide separate gross and microscopic dimensions as this causes confusion Clinical, pathologic, and radiologic assessment can all be used with pathology being the ultimate arbiter of tumor size Clinical examination should be used to stage if pathology and radiology are not available

### Multiple Specimens

It is often the case that the patient undergoes multiple procedures—loop excision(s), cone(s), trachelectomy, and hysterectomy with tumor in more than 1 specimen. In these situations, the important question arises of how best to measure tumor size and depending on the method used this may result in a different stage being assigned with important management implications. Some (eg, the ICCR) 1 advocate adding all maximum horizontal dimensions from each specimen, while others recommend using the largest horizontal measurement in any one specimen. Each situation poses its own problems. If adding all horizontal dimensions across multiple specimens, it is not possible to accurately align where 1 edge of the tumor from 1 specimen lines up with the correct edge on a different specimen. This will almost certainly result in an overestimation of tumor size.

Given that the new FIGO staging no longer uses horizontal extent as a criterion for staging microscopic tumors, this is perhaps less of an issue. If only the largest horizontal dimension in any 1 specimen is used, this may underestimate the maximum horizontal dimension. Horizontal extent can be reported as additive across multiple specimens, recognizing that this likely results in overestimating tumor size, or only the current size can be reported with a comment on the size in prior specimens. This also applies to multifocal tumors across multiple specimens, as can be seen in adenocarcinoma (see multifocal section below). In such cases, discussion at the tumor board/multidisciplinary team meeting should determine the final tumor stage. It is recommended that depth of invasion should be reported as measured on each specimen but for final staging purposes, the deepest invasion in any single specimen should be used.

**Table:** 

Recommendation for tumor measurement: multiple specimens
Depth of invasion should be reported as measured on each specimen but for final staging purposes, the deepest invasion in any single specimen should be used

### Exophytic Tumors

**FIG. 1:**
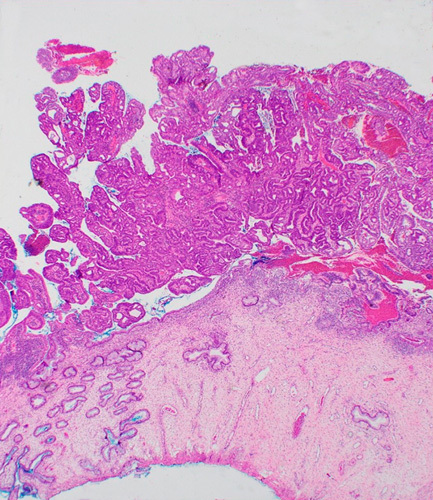
Exophytic cervical adenocarcinoma with minimal superficial stromal infiltration. Tumor thickness should be measured and if >5 mm, be used to upstage an otherwise IA tumor.

In purely or predominantly exophytic adenocarcinomas, the tumor can be quite large yet have minimal cervical wall infiltration (Fig. 1). This results in 2 separate dimensions: (1) tumor thickness as measured from the top-most part of the tumor to the bottom-most tip of the infiltrating front or base of the tumor if there is no stromal infiltration; this may constitute the maximum tumor dimension but does NOT equate to invasive depth, and (2) depth of invasion measured from the normal epithelial-stromal junction to the base of the infiltrating front if stromal infiltration is present. In the survey conducted by ISGyP, there was marked variability with more pathologists reporting the actual depth of invasion (only the invasion in the cervical wall) than the tumor thickness with or without the depth of invasion.

As recommended by FIGO, both maximum tumor dimension and depth of stromal infiltration should be provided in the pathology report, with a comment detailing how each measurement was derived. For exophytic tumors with cervical wall infiltration of ≤5 mm (stage IA), but tumor thickness >5 mm, we recommend basing the final FIGO stage on the maximum tumor thickness, that is stage IB. Recording both measurements will facilitate the accrual of information regarding the clinical importance of tumor thickness and depth of invasion in these uncommon neoplasms. The thickness of the cervical wall in the area of deepest invasion should also be provided. Although we recommend staging such tumors based on the thickness and equating it with depth of invasion for staging purposes, the clinical significance should be discussed at the tumor board/multidisciplinary team meeting; although there is limited evidence thus far, tumors with minimal stromal invasion may not have the same metastatic potential as tumors of similar thickness invading the stroma 5.

**Table:** 

Recommendation for tumor measurement: exophytic tumors
Both the largest tumor dimension and depth of stromal infiltration should be provided in the pathology report, with a comment detailing how each measurement was derived Total cervical wall thickness in the area of deepest invasion should be reported Exophytic tumors should be staged based on the largest tumor dimension, even if superficially invasive (≤5 mm); if the thickness is >5 mm, the tumor is staged as IB

### Multifocal Tumors

Multifocal carcinomas are invasive tumor foci that occur discontinuously, separated by uninvolved stroma. The distance between invasive foci that constitutes multifocality is not clearly defined or agreed upon, but some have suggested any foci >2 mm apart (the blocks should be leveled to ensure that the separate foci do not join) should be considered multifocal; these criteria have been endorsed by the ICCR 1,6,7. The ISGyP survey results show how varied pathologists are in determining multifocality. Some use 2 mm separation, others 5 mm, while still others require the involvement of different quadrants or cervical lips. Anecdotally, multifocality is less common in cervical adenocarcinomas as compared with squamous cell carcinomas and with the new FIGO system eliminating the horizontal extent as part of the staging, it may have different clinical implications.

Without specific studies in adenocarcinoma, one must extrapolate from the squamous cell carcinoma literature. In two small, retrospective studies evaluating outcomes in multifocal squamous cell carcinomas defined as being at least 2 mm apart, Day et al. 6 and McIlwaine et al. 7 found that there were no recurrences or metastases in a total of 47 cases with median follow-up periods of 7 yr and 45.5 mo, respectively, after excisions with clear margins. McIlwaine and colleagues pointed out that half of their cases would have been upstaged to IB1 had the tumors been measured contiguously rather than as separate foci. This provides some evidence for eliminating horizontal extent for staging purposes in microscopic disease. Based on these limited findings and the revised FIGO staging, the recommendation is in line with that of the ICCR 1 which is to measure each invasive focus individually if they are (1) located in different blocks separated by intervening uninvolved blocks; (2) located on separate cervical lips with discontinuous tumor (not involving the curvature of the canal); (3) situated apart from each other in the same section using 2 mm distance between the invasive foci. Only the depth of invasion should be used for staging purposes with the deepest invasion reported and the tumor staged accordingly. An important point is leveling blocks to ensure that the multiple foci do not “join up” to form a contiguous mass. It should also be noted than the 2 mm designation is completely arbitrary and this is an area which requires more study. Multiple specimens with multifocal disease should be treated similarly.

**Table:** 

Recommendation for tumor measurement: multifocal tumors
Each invasive focus should be measured individually if they are: Located in different blocks separated by intervening uninvolved blocks Located on separate cervical lips with discontinuous tumor (not involving the curvature of the canal) Situated at least 2 mm apart in the same section

### How Many Tumor Dimensions Should Be Reported?

Regarding the number of tumor dimensions to be reported, as per FIGO recommendations, most pathologists report at least 2 dimensions—the deepest invasion and the largest tumor dimension. This provides adequate information for tumor staging which best informs treatment planning. The third dimension can still be reported if local protocols mandate this (and is currently recommended by the ICCR) but this is unnecessary since tumor volume is not routinely taken into account in patient management.

Circumferential tumors that result in a “barrel” shaped cervix can be difficult to measure since they are sometimes not grossly visible yet involve all quadrants. Measuring each section with tumor in a linear manner would grossly overestimate tumor size; therefore, it is recommended that such tumors be measured grossly if possible and if not, the diameter of the cervix be used as the closest approximation of tumor size in these situations. This only applies to tumors that invade the full thickness of the cervical wall and involve all quadrants. In tumors that do not invade the full thickness of the cervix and/or do not involve all quadrants, only the depth of cervical wall infiltration and the largest tumor dimension as measured histologically should be provided, with a comment on the extent of tumor (eg, how many quadrants are involved) and the absence of a grossly visible tumor to measure. Occasionally, radiology may provide the most accurate tumor size and should be incorporated into final staging when appropriate.

In addition to reporting the absolute depth of invasion, the total thickness of the cervical wall in the area of deepest invasion should be reported in conjunction such that the percentage of stromal infiltration can be assessed and the presence of tumor within the inner, middle or outer third of the cervical stroma determined. This is to help guide the use of Sedlis criteria for adjuvant external pelvic radiation therapy following radical hysterectomy based on certain high-risk features (lymphovascular invasion, depth of stromal invasion by thirds, tumor size) 8.

**Table:** 

Recommendation for tumor measurement: how many dimensions to report
At least 2 measurements Depth of invasion Largest tumor dimension For circumferential “barrel” shaped cervix without grossly visible tumor: If tumor is in every quadrant with full thickness invasion of cervical wall, measure the diameter of cervix as closest approximation of tumor size For tumors without full thickness invasion and/or tumor in every quadrant, report the deepest invasion, largest tumor dimension as measured histologically and number of quadrants involved with a comment regarding lack of grossly measurable tumor Provide entire cervical wall thickness in area of deepest invasion (to calculate % depth of invasion)

### Microinvasive Carcinoma

The term “microinvasive carcinoma” does not appear in the FIGO staging system for cervical cancer. Furthermore, use of the term “microinvasive carcinoma” has different connotations in different geographical areas. For example, in the United Kingdom, microinvasive carcinoma was considered to be synonymous with FIGO stage IA1 and IA2 disease in most, but not all, institutions (some used the term “microinvasive carcinoma” to denote only FIGO stage IA1 tumors). Thus, in order to avoid confusion, it is recommended to avoid using the term “microinvasive carcinoma” but to accurately measure the tumor and use the specific FIGO or TNM stage.

**Table:** 

Recommendation for tumor measurement: “microinvasive carcinoma”
Do NOT use the term “microinvasive carcinoma” Measure the tumor as accurately as possible and use the specific FIGO or TNM stage

### Measurement of Tumors that Are a Combination of AIS and Adenocarcinoma

The assessment of tumors that are a combination of AIS and adenocarcinoma where it is difficult to delineate the 2 components should follow the guidance in another review in the series (Alvarado-Cabrero). Measurement of these lesions is somewhat analogous to that of exophytic tumors and should incorporate maximum horizontal tumor dimension and an assessment of invasive depth as accurately as possible, with these measurements forming the basis of staging. Where necessary this may be accompanied by a comment detailing the diagnostic problems; the distinction of *in situ* from invasive disease in such cases can be extremely subjective and problematic and this is an area where referral for a specialist opinion may be useful.

**Table:** 

Recommendation for tumor measurement: lesions which are combination of AIS and adenocarcinoma
Should incorporate maximum horizontal tumor dimension and assessment of invasive depth as accurately as possible

### Tumor at Margins of Excision Specimen

When tumor is present at the margins of a loop or cone excision, the true size of the tumor cannot be assessed accurately. In the new FIGO system, there is a statement that tumors that reach the margin should be staged as IB based on the positive margin. This was queried by ICCR and FIGO conceded that in these cases a repeat excision would be required to accurately assign a stage to the cancer. Therefore, it is recommended tumors should not be staged IB based only on positive margins. In most such cases, there will be a subsequent excision specimen and the stage should be determined based on the findings in all specimens. As an alternative, one can give a provisional stage, for example “at least stage x based on the measurements of the incomplete excision.”

**Table:** 

Recommendation for tumor measurement: staging of tumor at margins
Tumors should not be staged IB based only on positive margins Tumors with positive margins should not be staged—in most such cases, there will be a subsequent excision specimen on which the stage can be determined An alternative is to give a provisional stage, for example “at least stage x based on the measurements of the incomplete excision”

## TUMOR EXTENSION OUTSIDE THE CERVIX

### Parametrial/Paracervical Soft Tissue Involvement

Extrauterine involvement by cervical cancer is usually stage II or higher (see the next section on Adnexal involvement) and parametrial involvement is stage IIB. As formally defined, the parametrium consists of the connective tissue surrounding branches of the hypogastric vessels during their course toward the uterus and vagina and are located lateral to the uterus. There are occasions where the tumor extends beyond the cervix in the anterior or posterior plane, which technically is not encompassed in the anatomic delineation of parametria. Since any tumor that invades beyond the uterus without involving the lower third of the vagina or extending to the pelvic side wall is considered stage II, any anterior or posterior extension of tumor beyond the cervix should be staged as IIB since it is analogous to parametrial involvement for management purposes. It should be noted that parametrial lymph node involvement should be designated as stage IIIC1 in the revised FIGO system.

**Table:** 

Recommendation for tumor outside the cervix: paracervical extension
Tumor involving the anterior or posterior paracervical tissue, including extension to bladder or bowel WITHOUT mucosal involvement, should be staged as IIB (as this is clinically treated as parametrial involvement) Parametrial lymph node involvement should be staged IIIC1 as they are considered pelvic lymph nodes

### Adnexal Involvement

FIGO 2018 explicitly states that ovarian involvement by cervical carcinoma does not change the stage. There are limited data on the prognostic implications of tubo-ovarian involvement, and it is a rare event but more common in adenocarcinoma than squamous cell carcinoma. In early stage cervical cancer, the incidence of metastases to the ovaries is <1% for squamous cell carcinoma and <5% for adenocarcinoma 9–11. In addition, since it is often associated with other high-risk factors, there are limited data on its impact on survival as an independent risk factor. One study by Shimada et al. 10 showed that patients with ovarian metastases had poor outcomes unrelated to FIGO stage and the presence of ovarian metastases did not correlate with lymph node involvement or parametrial invasion, suggesting it may be an independent prognostic factor. It is likely that the clinical and prognostic implications of adnexal involvement differ between HPV-associated and HPV-independent cervical adenocarcinomas, and also depending on the route of spread and the pattern/extent of involvement 12–14.

There still are not enough robust data and in line with the FIGO directive, it is recommended to report ovarian and tubal involvement but not to alter stage based solely on their involvement. It is also the recommendation to specify the pattern of involvement in the fallopian tubes (mucosal epithelial, mucosal stromal, mural, serosal) as this will allow for prospective data collection for future studies to assess their impact on outcomes.

**Table:** 

Recommendation for tumor outside the cervix: adnexal involvement
Ovarian involvement does not upstage cervical carcinoma, but this should be documented on the pathology report Fallopian tube involvement does not upstage cervical carcinoma, but this should be documented on the pathology report; the location of tubal involvement should be documented (mucosal epithelial, mucosal stromal, mural, serosal or intravascular)

## LYMPHOVASCULAR SPACE INVASION (LVSI)

**FIG. 2:**
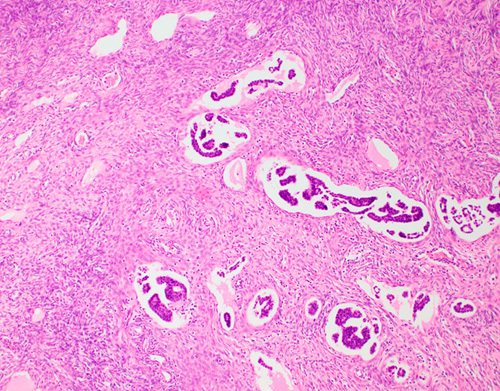
Lymphovascular space invasion of cervical adenocarcinoma in ovarian stroma. Ovarian involvement by cervical carcinoma does not change the stage, whether in vascular spaces or in ovarian parenchyma; however, it should always be reported as it may affect treatment.

FIGO 2018 addresses the issue of LVSI in parametrial tissue and states that tumors should not be upstaged based only on parametrial vascular invasion. In general, when tumor is present only in vessels outside the cervix (eg, parametrial, adnexal, etc.), this should not be considered involvement of that tissue and should not alter the stage, although it should be mentioned in the pathology report (Fig. 2). The issue of quantifying the number of involved vessels has been a recent topic of interest in gynecologic pathology, particularly in endometrial carcinomas where extent of LVSI has been found to be a strong independent prognostic factor for recurrence and overall survival 15–18. Comparable, but less numerous, studies in cervical cancer have shown the importance of LVSI in outcome, although quantification of LVSI was not generally studied 19–22. One study by Alvarado-Cabrero et al. 23 evaluating micropapillary architecture in cervical adenocarcinomas (a marker of aggressive behavior) quantified LVSI as negative, low (1–4 spaces involved), moderate (5–19 spaces involved) and extensive (≥20 spaces involved) and found significant association with overall survival (100%, 51.9%, and 0% for low, moderate, and extensive LVSI, respectively). However, micropapillary type endocervical adenocarcinomas are aggressive neoplasms which characteristically exhibit extensive LVSI and it remains to be proven in future studies if quantifying LVSI is clinically significant in cervical carcinoma, including adenocarcinoma, and what values, if any, should be used as cut-offs. It is recommended at this time that LVSI be reported as present or absent, but that quantification is not necessary, although this can be done locally which may facilitate accrual of information for future studies.

**Table:** 

Recommendation for LVSI
The presence or absence of LVSI should be included in the pathology report. LVSI at any location, including that seen outside the uterus (e.g. parametrial, adnexal) does not upstage a tumor.

## LYMPH NODES

One of the major changes to the revised FIGO 2018 system is the incorporation of lymph node involvement in assigning stage. It allows for assessment of retroperitoneal lymph nodes by imaging and/or pathologic findings and if deemed metastatic, is designated stage IIIC1 (pelvic/parametrial lymph node involvement) or IIIC2 (para-aortic lymph node involvement).

Sentinel lymph nodes are now commonly removed in cervical carcinoma and have been incorporated into the NCCN guidelines 24–28. Tumor involvement of lymph nodes, in line with the recommendations in TNM8, is reported as: negative, isolated tumor cells (ITCs, ≤0.2 mm), micrometastases (>0.2 and ≤2.0 mm), and macrometastases (>2 mm). Studies have shown that the presence of micrometastases in patients with early stage cervical cancer is significantly associated with reduced overall survival, equivalent to macrometastases, while no prognostic significance has generally been found for ITCs 29,30.

A statement in the original publication of the revised FIGO staging stated that the presence of micrometastases or ITCs may be recorded but “their presence does not change the stage.” The ICCR group queried FIGO regarding the designation of micrometastases in the same category as ITCs and FIGO did respond that this was an error and made the corrections in the subsequent corrigendum. In line with other organ systems, it is recommended that the presence of ITCs should be recorded but this does not count as nodal involvement, and should not result in tumor upstaging, designated pN0(i+) in TNM.

The issue of how to measure low-volume metastases in lymph nodes can be problematic. There are occasions where single cells and small clusters (≤0.2 mm) are present scattered throughout the entire lymph node (and therefore spans >0.2 mm in aggregate) yet the tumor foci are not contiguous suggesting they are not the same volumetrically as solid metastatic foci. The recommendation is to measure only contiguous tumor cells to designate ITCs and not aggregate all scattered clusters into a single measurement; however, if multiple collections of ITCs are present, this should be documented in the pathology report. The number of lymph nodes harboring ITCs/micro/macrometastases should also be recorded. When there is nodal involvement, the presence or absence of extracapsular/extranodal spread should be documented 31.

**Table:** 

Recommendation for sentinel lymph nodes
The presence of isolated tumor cells (ITCs) in lymph nodes should be recorded but this does NOT count as nodal involvement and should not result in tumor upstaging The number of lymph nodes harboring ITCs/micro/macrometastases should be recorded When ITCs are scattered throughout the lymph node, measure only contiguous tumor cells to designate ITCs and not aggregate all scattered clusters into a single measurement; however, if multiple collections of ITCs are present, this should be documented in the pathology report. When there is lymph node involvement, the presence or absence of extracapsular spread should be documented.

## CONCLUSIONS

The revised FIGO 2018 staging system includes major changes which better incorporate clinical, pathologic, and radiologic data, as well as including lymph node involvement in assigning stage. The horizontal measurement in microscopic disease is no longer a factor in assigning stage, which may have alleviated some of the problematic issues in tumor measurements. There are still controversial issues in pathologic reporting of cervical cancer, including adenocarcinoma, and we have provided recommendations for best practice based on the available evidence and sometimes on expert opinion. It is hoped that the ISGyP international data collection initiative will provide provisional answers to some of the remaining questions and form the basis for future prospective validation. It is further hoped that in future revisions of the FIGO staging system, international pathology organizations, such as ISGyP, will be more actively involved.

## References

[1] McCluggage WG Judge MJ Alvarado-Cabrero I. Data Set for the Reporting of Carcinomas of the Cervix: Recommendations From the International Collaboration on Cancer Reporting (ICCR). Int J Gynecol Pathol 2018;37:205–28. 28700433 10.1097/PGP.0000000000000412

[2] Bhatla N Berek JS Cuello Fredes M. Revised FIGO staging for carcinoma of the cervix uteri. Int J Gynaecol Obstet 2019;145:129–35. 30656645 10.1002/ijgo.12749

[3] Corrigendum to “Revised FIGO staging for carcinoma of the cervix uteri” [Int J Gynecol Obstet 145(2019) 129-135]. Int J Gynaecol Obstet 2019;147:279–80. 31571232 10.1002/ijgo.12969

[4] Salvo G Odetto D Pareja R. Revised 2018 International Federation of Gynecology and Obstetrics (FIGO) cervical cancer staging: a review of gaps and questions that remain. Int J Gynecol Cancer 2020;30:873–8. 32241876 10.1136/ijgc-2020-001257

[5] Trimbos JB Lambeek AF Peters AA. Prognostic difference of surgical treatment of exophytic versus barrel-shaped bulky cervical cancer. Gynecol Oncol 2004;95:77–81. 15385113 10.1016/j.ygyno.2004.06.025

[6] Day E Duffy S Bryson G. Multifocal FIGO Stage IA1 squamous carcinoma of the cervix: criteria for identification, staging, and its good clinical outcome. Int J Gynecol Pathol 2016;35:467–74. 26863478 10.1097/PGP.0000000000000269

[7] McIlwaine P Nagar H McCluggage WG. Multifocal FIGO stage 1A1 cervical squamous carcinomas have an extremely good prognosis equivalent to unifocal lesions. Int J Gynecol Pathol 2014;33:213–7. 24681729 10.1097/PGP.0b013e31829040ce

[8] Sedlis A Bundy BN Rotman MZ. A randomized trial of pelvic radiation therapy versus no further therapy in selected patients with stage IB carcinoma of the cervix after radical hysterectomy and pelvic lymphadenectomy: a Gynecologic Oncology Group Study. Gynecol Oncol 1999;73:177–83. 10329031 10.1006/gyno.1999.5387

[9] Landoni F Zanagnolo V Lovato-Diaz L. Ovarian metastases in early-stage cervical cancer (IA2-IIA): a multicenter retrospective study of 1965 patients (a Cooperative Task Force study). Int J Gynecol Cancer 2007;17:623–8. 17309669 10.1111/j.1525-1438.2006.00854.x

[10] Shimada M Kigawa J Nishimura R. Ovarian metastasis in carcinoma of the uterine cervix. Gynecol Oncol 2006;101:234–7. 16300819 10.1016/j.ygyno.2005.10.004

[11] Zhou J Chen Y Zhang P. Ovarian preservation in adenocarcinoma of the uterine cervix. J Ovarian Res 2017;10:48–52. 28738842 10.1186/s13048-017-0339-y PMC5525268

[12] Casey L Singh N. Metastases to the ovary arising from endometrial, cervical and fallopian tube cancer: recent advances. Histopathology 2020;76:37–51. 31846521 10.1111/his.13985

[13] Ronnett BM Yemelyanova AV Vang R. Endocervical adenocarcinomas with ovarian metastases: analysis of 29 cases with emphasis on minimally invasive cervical tumors and the ability of the metastases to simulate primary ovarian neoplasms. Am J Surg Pathol 2008;32:1835–53. 18813124 10.1097/PAS.0b013e3181758831

[14] Park KJ. Cervical adenocarcinoma: integration of HPV status, pattern of invasion, morphology and molecular markers into classification. Histopathology 2020;76:112–27. 31846527 10.1111/his.13995

[15] Bosse T Peters EE Creutzberg CL. Substantial lymph-vascular space invasion (LVSI) is a significant risk factor for recurrence in endometrial cancer—a pooled analysis of PORTEC 1 and 2 trials. Eur J Cancer 2015;51:1742–50. 26049688 10.1016/j.ejca.2015.05.015

[16] Hachisuga T Kaku T Fukuda K. The grading of lymphovascular space invasion in endometrial carcinoma. Cancer 1999;86:2090–7. 10570436

[17] Kuroki J Hasegawa K Kato R. Relationship between the classification of vascular invasion severity and the prognosis of uterine endometrial cancer. Int J Gynecol Cancer 2003;13:47–52. 12631220 10.1046/j.1525-1438.2003.13023.x

[18] Winer I Ahmed QF Mert I. Significance of lymphovascular space invasion in uterine serous carcinoma: what matters more; extent or presence? Int J Gynecol Pathol 2015;34:47–56. 25473753 10.1097/PGP.0000000000000113

[19] Roma AA Park KJ Xie H. Role of lymphovascular invasion in pattern C invasive endocervical adenocarcinoma. Am J Surg Pathol 2017;41:1205–11. 28614201 10.1097/PAS.0000000000000822

[20] Creasman WT Kohler MF. Is lymph vascular space involvement an independent prognostic factor in early cervical cancer? Gynecol Oncol 2004;92:525–9. 14766243 10.1016/j.ygyno.2003.11.020

[21] Memarzadeh S Natarajan S Dandade DP. Lymphovascular and perineural invasion in the parametria: a prognostic factor for early-stage cervical cancer. Obstet Gynecol 2003;102:612–9. 12962952 10.1016/s0029-7844(03)00569-6

[22] Morice P Piovesan P Rey A. Prognostic value of lymphovascular space invasion determined with hematoxylin-eosin staining in early stage cervical carcinoma: results of a multivariate analysis. Ann Oncol 2003;14:1511–7. 14504051 10.1093/annonc/mdg412

[23] Alvarado-Cabrero I McCluggage WG Estevez-Castro R. Micropapillary cervical adenocarcinoma: a clinicopathologic study of 44 cases. Am J Surg Pathol 2019;43:802–9. 30864975 10.1097/PAS.0000000000001245 PMC8258798

[24] Cibula D Abu-Rustum NR Dusek L. Bilateral ultrastaging of sentinel lymph node in cervical cancer: lowering the false-negative rate and improving the detection of micrometastasis. Gynecol Oncol 2012;127:462–6. 22943880 10.1016/j.ygyno.2012.08.035

[25] Lécuru F Mathevet P Querleu D. Bilateral negative sentinel nodes accurately predict absence of lymph node metastasis in early cervical cancer: results of the SENTICOL study. J Clin Oncol 2011;29:1686–91. 21444878 10.1200/JCO.2010.32.0432

[26] Lennox GK Covens A. Can sentinel lymph node biopsy replace pelvic lymphadenectomy for early cervical cancer? Gynecol Oncol 2017;144:16–20. 27742472 10.1016/j.ygyno.2016.08.337

[27] Tax C Rovers MM de Graaf C. The sentinel node procedure in early stage cervical cancer, taking the next step; a diagnostic review. Gynecol Oncol 2015;139:559–67. 26416173 10.1016/j.ygyno.2015.09.076

[28] Wang XJ Fang F Li YF. Sentinel-lymph-node procedures in early stage cervical cancer: a systematic review and meta-analysis. Med Oncol 2015;32:385. 25429838 10.1007/s12032-014-0385-x PMC4246132

[29] Cibula D Abu-Rustum NR Dusek L. Prognostic significance of low volume sentinel lymph node disease in early-stage cervical cancer. Gynecol Oncol 2012;124:496–501. 22120175 10.1016/j.ygyno.2011.11.037

[30] Cibula D McCluggage WG. Sentinel lymph node (SLN) concept in cervical cancer: current limitations and unanswered questions. Gynecol Oncol 2019;152:202–7. 30318103 10.1016/j.ygyno.2018.10.007

[31] Cibula D Potter R Planchamp F. The European Society of Gynaecological Oncology/European Society for Radiotherapy and Oncology/European Society of Pathology Guidelines for the management of patients with cervical cancer. Int J Gynecol Cancer 2018;28:641–55. 29688967 10.1097/IGC.0000000000001216

